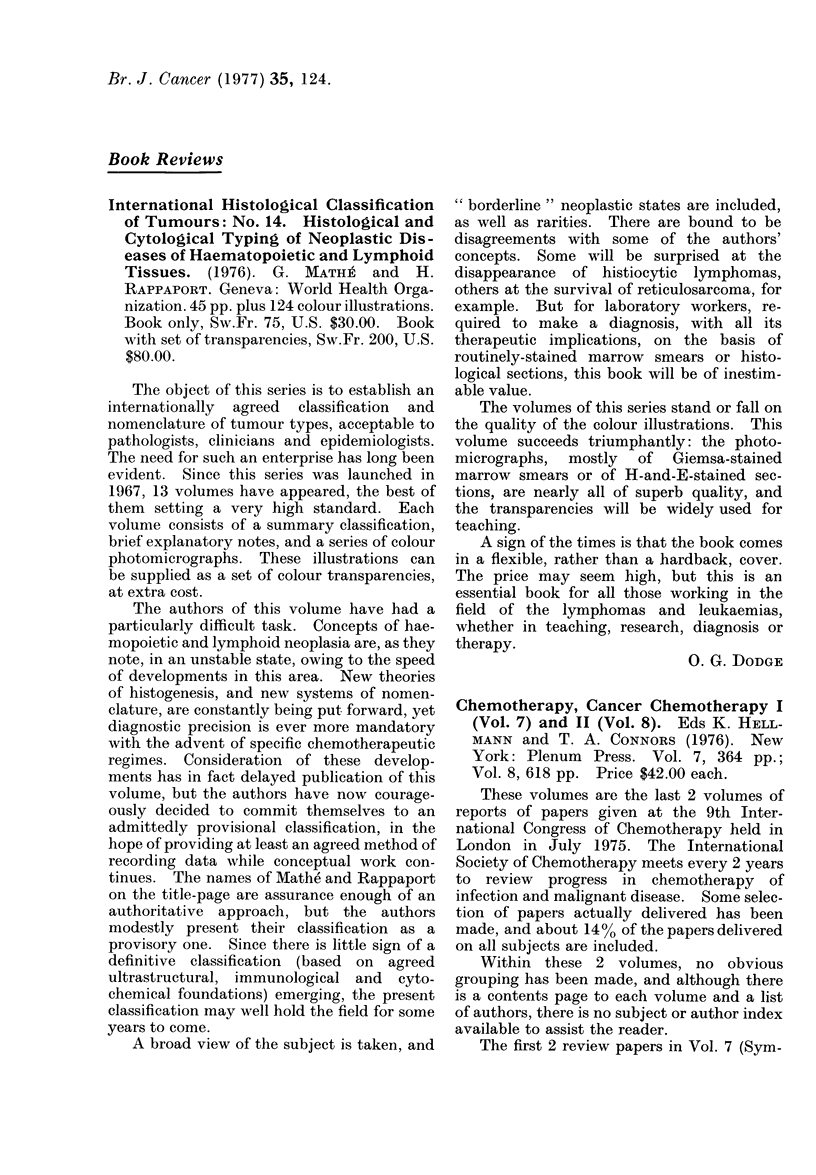# International Histological Classification of Tumours: No. 14. Histological and Cytological Typing of Neoplastic Diseases of Haematopoietic and Lymphoid Tissues

**Published:** 1977-01

**Authors:** O. G. Dodge


*Br. J. Cancer* (1977) **35**, 124.

### *Book Reviews*

**International Histological Classification of Tumours: No. 14. Histological and Cytological Typing of Neoplastic Diseases of Haematopoietic and Lymphoid Tissues.** (1976). G. MATHÉ and H. RAPPAPORT. Geneva: World Health Organization. 45 pp. plus 124 colour illustrations. Book only, Sw.Fr. 75, U.S. $30.00. Book with set of transparencies, Sw.Fr. 200, U.S. $80.00.

The object of this series is to establish an internationally agreed classification and nomenclature of tumour types, acceptable to pathologists, clinicians and epidemiologists. The need for such an enterprise has long been evident. Since this series was launched in 1967, 13 volumes have appeared, the best of them setting a very high standard. Each volume consists of a summary classification, brief explanatory notes, and a series of colour photomicrographs. These illustrations can be supplied as a set of colour transparencies, at extra cost.

The authors of this volume have had a particularly difficult task. Concepts of haemopoietic and lymphoid neoplasia are, as they note, in an unstable state, owing to the speed of developments in this area. New theories of histogenesis, and new systems of nomenclature, are constantly being put forward, yet diagnostic precision is ever more mandatory with the advent of specific chemotherapeutic regimes. Consideration of these developments has in fact delayed publication of this volume, but the authors have now courageously decided to commit themselves to an admittedly provisional classification, in the hope of providing at least an agreed method of recording data while conceptual work continues. The names of Mathè and Rappaport on the title-page are assurance enough of an authoritative approach, but the authors modestly present their classification as a provisory one. Since there is little sign of a definitive classification (based on agreed ultrastructural, immunological and cytochemical foundations) emerging, the present classification may well hold the field for some years to come.

A broad view of the subject is taken, and " borderline " neoplastic states are included, as well as rarities. There are bound to be disagreements with some of the authors' concepts. Some will be surprised at the disappearance of histiocytic lymphomas, others at the survival of reticulosarcoma, for example. But for laboratory workers, required to make a diagnosis, with all its therapeutic implications, on the basis of routinely-stained marrow smears or histological sections, this book will be of inestimable value.

The volumes of this series stand or fall on the quality of the colour illustrations. This volume succeeds triumphantly: the photomicrographs, mostly of Giemsa-stained marrow smears or of H-and-E-stained sections, are nearly all of superb quality, and the transparencies will be widely used for teaching.

A sign of the times is that the book comes in a flexible, rather than a hardback, cover. The price may seem high, but this is an essential book for all those working in the field of the lymphomas and leukaemias, whether in teaching, research, diagnosis or therapy.

O. G. DODGE